# Effect of Ethylenediaminetetraacetic Acid on Unsaturated Poly(Butylene Adipate-*Co*-Butylene Itaconate) Copolyester with Low-Melting Point and Controllable Hardness

**DOI:** 10.3390/polym11040611

**Published:** 2019-04-03

**Authors:** Chin-Wen Chen, Te-Sheng Hsu, Syang-Peng Rwei

**Affiliations:** 1 Institute of Organic and Polymeric Materials, National Taipei University of Technology, No. 1, Sec. 3, Chung-Hsiao East Road., Taipei 10608, Taiwan; cwchen@ntut.edu.tw (C.-W.C.); hsutesheng1991@gmail.com (T.-S.H.); 2 Research and Development Center of Smart Textile Technology, National Taipei University of Technology, No. 1, Sec. 3, Chung-Hsiao East Road., Taipei 10608, Taiwan

**Keywords:** poly(butylene adipate), poly(butylene itaconate), ethylenediaminetetraacetic acid, low-melting point polyester, partially cross-linking, thermoplastic, controllable hardness, smart textile

## Abstract

A series of copolyesters, poly(butylene adipate-*co*-butylene itaconate) (PBABI), was synthesized using melt polycondensation from adipic acid (AA), itaconic acid (IA), 1,4-butanediol (1,4-BDO), and ethylenediaminetetraacetic acid (EDTA). ^1^H-NMR, FT-IR, GPC, DSC, TGA, DMA, XRD, Shore D, and tensile test were used to systematically characterize the structural and composition/physical properties of the copolyesters. It was found that the melting point (*T*_m_) and crystallization temperature (*T*_c_) of the copolyesters were, respectively, between 21.1 to 57.5 °C and −6.7 to 29.5 °C. The glass transition (*T*_g_) and the initial thermal decomposition (*T*_d-5%_) temperatures of the PBABI copolyesters were observed to be between −53.6 to −55.8 °C and 313.6 and 342.1 °C at varying ratios of butylene adipate (BA) and butylene itaconate (IA), respectively. The XRD feature peak was identified at the 2θ values of 21.61°, 22.31°, and 23.96° for the crystal lattice of (110), (020), and (021), respectively. Interestingly, Shore D at various IA ratios had high values (between 51.3 to 62), which indicated that the PBABI had soft plastic properties. The Young’s modulus and elongation at break, at different IA concentrations, were measured to be at 0.77–128.65 MPa and 71.04–531.76%, respectively, which could be attributed to a close and compact three-dimensional network structure formed by EDTA as a crosslinking agent. There was a significant bell-shaped trend in a BA/BI ratio of 8/2, at different EDTA concentrations—the ∆*H*_m_ increased while the EDTA concentration increased from 0.001 to 0.05 mole% and then decreased at an EDTA ratio of 0.2 mole%. Since the PBABI copolymers have applications in the textile industry, these polymers have been adopted to reinforce 3D air-permeable polyester-based smart textile. This kind of composite not only possesses the advantage of lower weight and breathable properties for textiles, but also offers customizable, strong levels of hardness, after UV curing of the PBABI copolyesters, making its potential in vitro orthopedic support as the “plaster of the future”.

## 1. Introduction

Polyester material, known as polyethylene terephthalate (PET), is one of the most critical commercial thermoplastic materials. It is a semi-crystalline polymer with excellent performance in its clarity, stress cracking, chemical resistance, thermal stability, melt mobility, spinning, and so forth [1]. For many years, PET has been adopted for man-made fibers, packaging material, optical film, electrical device packaging, automotive, and geotextiles. Most PET production is consumed by the clothing and technological textile fiber manufacturing industry.

Polyester material is usually formed through melt polymerization of diacid or its derivatives with diols, in industrial mass production. In this field, PET delivers many unique benefits over polymers like polypropylene terephthalate (PTT) [2], polybutylene terephthalate (PBT) [3], polyhexamethylene terephthalate (PHT) [4], and polyethylene naphthalate (PEN) [5], such as anti-UV properties and a gas barrier with the PEN resin. These kinds of polyester consist of a terephthalate or naphthalate ring within a repeating unit, which can have a higher melting point and greater mechanical strength.

Aromatic polyester is not suitable as a low-melting point polyester [6,7,8,9] for coating and fiber applications. Aliphatic polyester could be leveraged because of its lower melting temperature, thermoplasticity, and biodegradability [10,11,12,13,14,15]. However, its suitability for commercial use is limited due to its poor mechanical characteristics. In contrast, unsaturated aliphatic polyester can be modified to form a network structure, by chemically cross-linking modifiers such as telechelic oligoesters and diisocyanates, diepoxides or triethoxysilanes units, to form thermoset-like copolyesters [16,17,18], or by combining it with epoxy materials [19,20]. This modification aims to obtain excellent physico-chemical properties that differ from the characteristic properties of polyesters, such as stronger hardness, higher rigidity, and better tensile strength and thermal stability [21].

One advantage of aliphatic polyester is the polymer’s potential to form a 3D network architecture, through copolymerization with multi-functional cross-linking agents [22,23], such as benzene-1,3,5-tricarboxylic acid and glycerol, in the tri-functional group; ethylenediaminetetraacetic acid, 2,2-Bis(hydroxymethyl)1,3-propanediol, and 1,2,4,5-Benzenetetracarboxylic acid in the tetra-functional group; and hexahydroxycyclohexane and cyclohexane-1,2,3,4,5,6-hexacarboxylic acid, in the hexa-functional group. All the multi-functional groups of cross-linking agents were tested in a strictly controlled molar mass, to investigate the thermal and mechanical properties in our research.

Aliphatic polyester is a semi-crystalline polymer and an important biodegradable material. Its chemical structure includes an ester and ether bond, which leads to a good flexibility in the polymer chain, mechanical properties, and biodegradability. Some popular aliphatic polyesters have been synthesized as polylactic acid (PLA) [24,25] through chemical methods, polycaprolactone (PCL) through ring-opening polymerization [26,27,28,29], polyhydroxybutyrate (PHB) [30,31] via bacterial synthesis (as poly(butylene succinate) (PBS)) [32,33,34], poly(propylene sebacate) (PPS) [35], poly(butylene adipate) (PBA) [36,37,38,39], poly(butylene itaconate) (PBI) [17,40,41], and their copolymers. Aliphatic copolyesters have also been synthesized to improve their physical properties. For example, Herrera et al. synthesized poly(butylene adipate-co-terephthalate) and analyzed their characteristics and degradation behavior. The thermal properties and Young’s modulus improved due to the terephthalate ring, and the enzymatic degradation property was maintained, even when the benzenic ring was copolymerized into the backbone of the molecular chain [42]. Simonida et al. copolymerized poly(itaconic acid)and poly(ethylene glycol), to enhance the density of hydrogen bonding and obtain a more ordered structure, to adopt in mucoadhesive applications [43]. Polybutylene itaconate for modification with divinylbenzene (DVB) and glycerol has been successfully copolymerized by Wibowo et al., to form a 3-D network structure to increase the thermal stability of the copolyester [44]. For biomass material, 2,5-furan dicarboxylic acid (FDCA), was used to synthesize the unsaturated copolyester with fumaric acid, succinic acid, 1,3-propanediol, and 2-hydroxyethyl methacrylate, as a cross-linking agent, to form a cross-linked resin that has a good thermal stability at 231 °C, and a higher *T*_g_ around 87–104 °C, due to the effect of the furan ring [45]. As fully bio-based unsaturated polyester has become a more popular research topic, Dai and colleagues considered fully bio-based unsaturated copolyesters consisting of FDCA and itaconic acid, which have a higher temperature of 5% thermal weight loss, tensile strength, and a Young’s modulus of 330 °C, 122.8 MPa, and 3521 MPa, respectively [46].

PBA is a typical polymorph aliphatic polyester. Therefore, the various crystal phases could be transformed in α-, β-, and an α-β complex, through an isothermal annealing process [38,39]. The α-phase is related to the thermodynamic stability, while the β-phase is a metastable phase and can be transformed into an α-phase, via annealing treatment. The α-phase of PBA achieved from the isothermal method, and annealing procedure has a different crystal morphology, size, and biodegradation behavior, due to the effect of the thermal history. Biodegradation ability is strongly related to heat treatment, and it is easy to investigate the structure–property relationship. Additionally, the unsaturated polyester, PBI, is a novel unsaturated polyester that is prepared from 1,4-butanediol (1,4-BDO) and biomass materials itaconic acid (IA), through conventional bulk melting polymerization [47]. IA is a dicarboxylic acid chemical, which is biomass, unsaturated, and sustainable material, and can be fermented with *Aspergillus Terreus.* It has been widely applied in adhesion resin applications [48,49]. The IA, succinic acid, and 1,4-BDO were copolymerized through solvent-free polycondensation, for bio-based, UV-cured coating application, which indicated that the mechanical property was a function of IA concentration [50]. Novel aliphatic copolyesters like poly(butylene adipate-co-butylene itaconate) (PBABI) [51], which has a relatively high molecular weight but poor mechanical properties, have additionally been studied. The cross-linking agents focus on copolymerization to improve the mechanical and thermal properties of copolyesters.

Ethylenediaminetetraacetic acid (EDTA) is a conventional chelating agent that is widely used to remove metal ion, antioxidant, textile, and biomedical applications [52,53]. Based on the chemical structure, the EDTA has four symmetrical carboxyl groups, which could react with the hydroxy group to form an ester bond. Copolymerizing with EDTA as a cross-linking agent can form network polymers to increase the toughness and elasticity of copolymers [54]. These kinds of network structures play a role in improving the thermal stability and mechanical strength of copolymers [55]. Hence, a small amount of EDTA was applied as a cross-linking agent to form a partial 3-D network structure in this study.

**Scheme 1:**
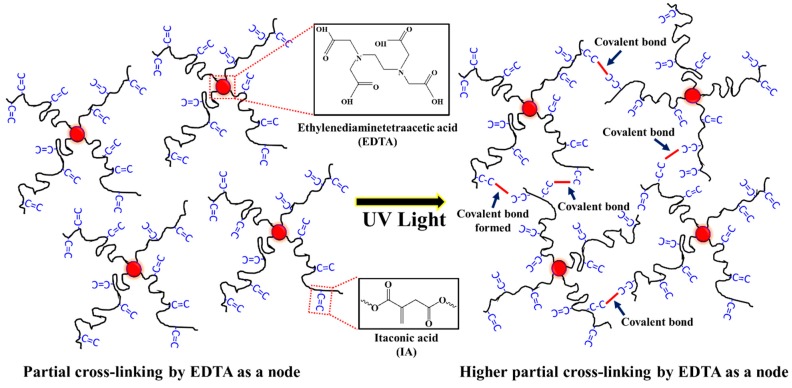
Thermoplastic poly(butylene adipate-*co*-butylene itaconate) (PBABI) copolyesters with UV curing.

Herein, the purpose of this research was to investigate the straightforward preparation of a series of PBABI copolyesters, based on AA, IA, and 1,4-BDO with EDTA, using titanium (IV) butoxide and dibutyltin dilaurate as the catalyst, via melting polymerization through a 2 L steel autoclave reactor. IA was adopted to achieve controllable hardness in the low-melting point PBABI copolyesters, following UV curing, due to the higher partial cross-linking formation, as shown in Scheme 1. The EDTA could form a 3D network structure to ensure thermal stability and maintain mechanical properties. In the first part of the research, the various components of AA and IA were held at a constant EDTA ratio of 0.1 mole%, to ensure a melting point range between 30–60 °C, which could be utilized in air-permeable 3D polyester fabric textiles, to deliver the mechanical strength and the supportive properties of the composite materials. The influence of varying ratios of EDTA between 0.001 to 0.2 mole% at a BA/BI ratio of 8/2 was also explored to observe changes in mechanical properties and find the appropriate EDTA concentration in the PBABI copolyester system.

## 2. Experiments

### 2.1. Materials

Itaconic acid (IA, 99%) and 1,4-butanediol (1,4-BDO, 99%) were purchased from the First Chemical Corporation (Taipei City, Taiwan). The adipic acid (AA, 99.8%) was supplied by the Asahi Kasei Corporation (Tokyo, Japan). The ethylenediaminetetraacetic acid (EDTA, 98%) was obtained from Vetec (St. Louis, MO, USA). The 4-Methoxyphenol (99%), titanium(IV) butoxide (T_i_(OB_u_)_4_, 97%), deuterium trifluoroacetic acid (d-TFA, 99.5%), and hexafluoroisopropanol (HFIP, 99%) were purchased from Aldrich (St. Louis, MO, USA). The dibutyltin dilaurate (DBTDL, 95%) was obtained from Alfa Aesar (Tewksbury, MA, USA). All chemicals were adopted in melting polymerization without any purification.

### 2.2. Sample Preparation

#### Synthesis of Poly(Butylene adipate-*co*-Butylene itaconate)s Copolyesters with Ethylenediaminetetraacetic Acid as a Cross-linking Agent

The PBABI copolyesters were copolymerized through melt polymerization in the one-pot reaction. First, AA and IA were esterified with 1,4-BDO, with EDTA, 4-Methoxyphenol, DBTDL, and T_i_(OB_u_)_4_ acting as a crosslinking agent, inhibitor, and catalyst, respectively. A 2 L steel reactor was used in melt copolymerization. The synthesized polyesters referred to as BA/BI = x/y correspond to the ratio of butylene adipate and butylene itaconate, where x and y are the mole percentages of AA and IA, respectively. The molar ratio of [OH]:[COOH] was kept at 1.2:1 in all PBABI copolyesters, and the different molar ratios of AA:IA were 10:0 (denoted as BA/BI = 10/0, also known as PBA), 9:1 (BA/BI = 9/1), 8:2 (BA/BI = 8/2), and 7:3 (BA/BI = 7/3). The 4-Methoxyphenol, dibutyltin dilaurate (DBTEL), and T_i_(OB_u_)_4_ were 0.5, 1, or 0.5 wt %, depending on the overall theoretical copolyester production. The concentration of the EDTA, with varying BA/BI ratios, was set at 0.1 mole%. To consider the effect of various EDTA concentrations, the BA/BI ratio was fixed at 8/2, with concentrations of 0.001, 0.01, 0.05, 0.1, and 0.2 mole%, for further discussion.

The detailed operating conditions of the 2 L reactor were as follows. The pressure of the reactor was maintained in 2 bars, with stirring at 80 rpm under nitrogen, for 4 h, to ensure the completion of esterification. The temperature chosen for the esterification stage was 190 °C, to prevent the 1,4-BDO from forming tetrahydrofuran (THF). The reflux temperature was set at 130 °C, to prevent the 1,4-BDO from being taken out by H_2_O. The degree of esterification was checked by the cool-condensed H_2_O to exceed 90%, with the calculated amount of H_2_O (calculated by the overall mole of diacid * molecular weight of H_2_O * 2). In the polycondensation stage, the temperature was held at 230 °C, for 2–3 h, under a high vacuum below 1.0 Torr. The torque value (Watt, W) before vacuum operation was chosen as the reference value. When the experimental torque value had reached 1.3–1.4 fold over the reference value, the copolymerization was selected to finish the reaction. Finally, the melted polymer was cooled into a solid state, using iced water, for further analysis and processing.

### 2.3. Measurements

#### 2.3.1. Nuclear Magnetic Resonance Spectroscopy (^1^H NMR)

A JEOL ECZ600R (600 MHz, Tokyo, Japan) ^1^H NMR spectrometer was used to study the chemical structure of the PBABI copolyesters. For each ^1^H NMR analysis, 100 mg of the synthesized copolyesters were dissolved in about 1 mL of trifluoroacetic acid-d (d-TFA, 99.5%). The solutions were then transferred into 5 mm ^1^H NMR sample tubes. The measurements were performed at room temperature (RT), and there were 128 recorded scans.

#### 2.3.2. Fourier Transform Infrared Spectroscopy (FT-IR)

Fourier transform infrared (FT-IR) spectroscopy measurement was carried out using a PerkinElmer Spectrum Two spectrometer (Waltham, MA, USA). The PBABI copolyesters were measured with an average signal of 32 co-added scans, at a resolution of 4 cm^−1^ in the wavenumber range 500–4000 cm^−1^. The spectra were received from the tablets of the PBABI copolyesters spread into KBr pellets, and the concentration was chosen to be 1% (150 mg).

#### 2.3.3. Gel Permeation Chromatography (GPC)

The PBABI copolyesters were dissolved in HFIP for 12 h and then filtered through a 0.2 μm PTFE filter membrane. The Viscotek GPC System (1122 pump, 2707 Auto-Injector, 270 LS Laser Light Scattering Detector/Viscometer, Shodex 71 RI detector, OmniSEC 4.6 Station, Malvern, UK) was used with the HFIP 806M Shodex column. The oven temperature, flow rate, and analysis time were set at RT, 1 mL·min^−1^ with HFIP, and 60 min, respectively.

#### 2.3.4. Intrinsic Viscosity (I.V.)

The intrinsic viscosity (I.V.) of the PBABI copolyesters (1.0 g·dL^−1^) in a mixture of phenol and tetrachloroethane (60/40, wt %) was measured, using an Ubbelodhe viscometer at 25 ± 0.05 °C. The I.V. values for the PBABI copolyesters are summarized in Table 2.

#### 2.3.5. Differential Scanning Calorimetry (DSC)

DSC (PerkinElmer DSC 800, Waltham, MA, USA) was used to measure the melting temperatures (*T*_m_), crystalline temperatures (*T*_c_), as well as melting enthalpies (Δ*H*_m_) and crystalline enthalpies (Δ*H*_c_) of the PBABI copolyesters. For each measurement, the PBABI copolyesters were set at a heating rate of 10 °C·min^−1^ from −150 to 100 °C, and kept at this temperature for 10 min, to remove any thermal history. Then, these samples were cooled to −150 °C at a cooling rate of 10 °C·min^−1^, for the first cycle. After this, the second cycle of the heating process, from −150 to 100 °C, at the same heating rate of 10 °C·min^−1^, was run to reach the melting point. The *T*_m_ and *T*_c_ of the copolyesters were determined from the maximum endothermic peak and exothermic peak, through the first cycle cooling and second cycle heating process, respectively. All DSC measurements were implemented in a nitrogen atmosphere with aluminum pans.

#### 2.3.6. Thermogravimetric Analysis (TGA)

Thermogravimetric analysis (TGA, STA 7200 HITACHI, Tokyo, Japan) was adopted to measure the degradation temperature and differential thermal gravity (DTG) of the PBABI copolyesters. The samples in the weight range of 5–10 mg were heated from 50 to 600 °C, at a heating rate of 10 °C·min^−1^, under a nitrogen atmosphere. The characteristic onset of the degradation temperature from the TGA curve was determined at 5% weight loss (*T*_d-5%_).

#### 2.3.7. Dynamic Mechanical Analysis (DMA)

The viscoelastic properties of the PBABI copolyesters were estimated using a dynamic mechanical analyzer (DMA, Tech Max DMS 6100, Tokyo, Japan), to obtain storage modulus (*E*’), loss modulus (*E*’’), and loss tangent (tan δ). The samples were tested in the compression mode with 150 mN, at a frequency of 1 Hz, and a temperature range from −150 to 0 °C, at a heating rate of 10 °C·min^−1^. The sample size tested was set at 30 mm (Length), 10 mm (Width), and 2 mm (Thickness).

#### 2.3.8. X-ray Diffraction (XRD)

The film of the PBABI copolyesters was prepared first, and then the X-ray diffraction pattern of the PBABI film was recorded using a Malvern Panalytical X’Pert^3^ powder diffractometer (Malvern, UK), with Cu Kα radiation (λ = 0.154 nm) in 2θ, from 10 to 40 degrees, with a scanning speed of 0.2°·min^−1^.

#### 2.3.9. UV Curing Procedures

A high-pressure mercury lamp with a power of 1,000 W at a wavelength of 365 nm and intensity of UV light of about 300 mW·cm^−2^, in a UVA range, was used for UV curing. The irradiation distance from the lamp to the surface of the PBABI copolyesters was carried out at 15 cm for 1, 2, 5, and 10 min.

#### 2.3.10. Tensile Test

The conventional hot-melt compression molding at a temperature of 80 °C and a pressure of 3 kg·cm^−2^ in 2 min, was performed, to prepare the dumb-bell shaped specimens of the PBABI copolyesters. Then, the measurements were taken at a crosshead speed of 50 mm·min^−1^, using Cometech QC-508M2F (Taichung City, Taiwan), based on the ASTM638 Type IV standard to obtain a stress–strain curve. The tensile strength, elongation at break, and Young’s modulus were evaluated from the stress–strain data. The average value of all the data was obtained on five specimens. The hardness of the samples was recorded using Shore hardness testers (SHAHE/LX-D, Taipei City, Taiwan), before and after various UV curing times. For each sample, the Shore D values were measured 10 times, to obtain the average values and standard error.

#### 2.3.11. Simulation Procedures of All-Atom Molecular Dynamics (AAMD)

The EDTA molecule as a center molecule formed four ester bonds with the 1,4-BDO, to investigate the rigidity of EDTA as a node, and then connected with three AA and one IA (named EDTA-BDO-BA/BI), for our all-atom simulation model. This model could be described as the chain movement to affect the geometric deviation of the cross-linking point, through torsional angle distribution analysis. The artificial model was built in a coplanar and tetrahedral geometrical structure, and then the torsional angle of N–C–C–N, within EDTA and N–C–C–O, between EDTA and 1,4-BDO, were analyzed to investigate the structural deviation [56]. All-atom molecular dynamics (AAMD) with a COMPASS force-field, was performed for a simulation model, to calculate the torsional angle distribution and to investigate the dynamic structure behavior. COMPASS force-field has shown robust performance in predicting structures and dynamics of organic molecules in AAMD simulations [57]. Thus, the probability distribution results obtained through the AAMD could illustrate the molecular chain behavior. The EDTA-BDO-BA/BI model was centered within the cubic box, with a side length of 100 Å, which was large enough to ensure an isolated molecular chain state. All simulations were adopted in the canonical ensemble (NVT), with a 1 fs time-step. The thermostat chosen was the Nose-Hoover method, with a Q ratio of 0.01 [58,59], and 15.5 Å was selected as the cutoff. The simulation temperatures were chosen based on the *T*_m_—those below *T*_m_ with 298 K, near *T*_m_ with 333 K, and above *T*_m_ with 373 K. First, the conjugate gradient geometry minimization method was used to relax the chain conformation and equilibrate the artificial structure. After the equilibration process, the dynamic simulations were performed in 1 ns, with a time-step of 1 fs at 298, 333, and 373 K, respectively. Finally, the molecular dynamics (MD) simulation was taken, and then the trajectory was analyzed through the averaged distribution functions of torsional angles in the N–C–C–N in EDTA and the N–C–C–O, between EDTA and 1,4-BDO, under various temperatures.

## 3. Results and Discussion

### 3.1. The Effect of the BA/BI Ratio of PBABA Copolyesters with an EDTA Concentration of 0.1 mole%

**Figure 1:**
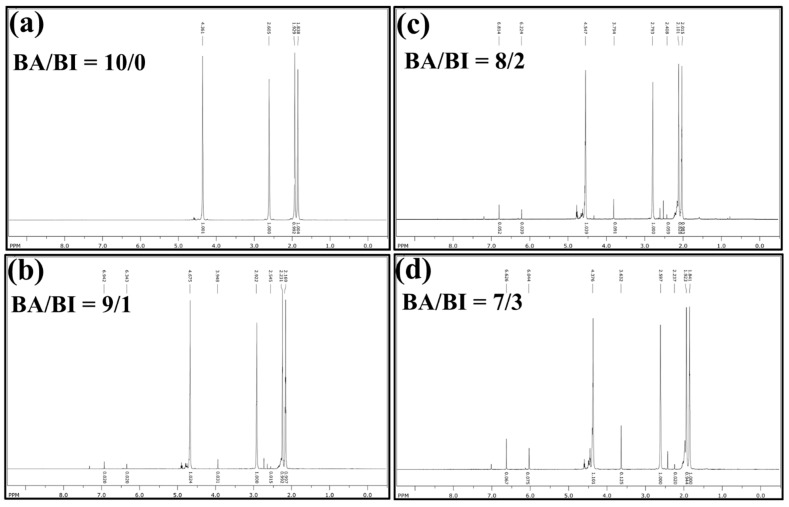
^1^H NMR spectra of PBABI copolyesters at (**a**) BA/BI = 10/0, (**b**) BA/BI = 9/1, (**c**) BA/BI = 8/2, and (**d**) BA/BI = 7/3.

The chemical structure and comonomer composition of PBABI copolyesters were first confirmed by NMR spectroscopy. The ^1^H NMR spectra of PBABI copolyesters are displayed in Figure 1. The resonance peaks of the PBABI copolyesters were identified and assigned in H1 (1.838–2.169 ppm, 3, 4-CH_2_ of adipic acid), H2 (1.923–2.231 ppm, 2, 3-CH_2_ of 1.4-butanediol), H3 (2.015–2.545 ppm, –CH_2_ between two nitrogen of Ethylenediaminetetraacetic acid), H4 (2,579–2.922 ppm, 2, 5-CH_2_ of adipic acid), H5 (3.632–3.948 ppm, 2-CH_2_ of itaconic acid), H6 (4.361–4.675 ppm, 1, 4-CH_2_ of 1.4-butanediol), H7 (6.044–6.343 ppm, –C=CH_2_ of itaconic acid), and H8 (6.626–6.942 ppm, –C=CH_2_ of itaconic acid). All individual values of resonance peaks, the integral ratio, and the calculated composition of IA, within the PBABI copolyesters, are summarized in Table 1. From the ratio of the integral area of ^1^H NMR resonance peaks of the PBABI copolyesters, the calculated rates of C=C of IA in BA/BI = 9/1, 8/2, and 7/3 were obtained at 4.51%, 11.41%, and 15.01%, because the C=C double-bond of the IA might isomerize into 2-methyl fumarate, which has a significantly lower reactivity and cannot be observed in NMR measurements [60]. Moreover, the catalyst selectivity in the esterification stage could cause the IA concentration to copolymerize into PBABI copolyesters [40,61]. The concentration of the C=C bond of IA can be maintained near 50%, in each PBABI copolyesters, through high-temperature melt polymerization, with the DBTEL catalyst, and T_i_(OB_u_)_4_, which could participate in the successive UV curing procedure, to adjust the hardness of the PBABI copolyesters through the UV curing time.

**Figure 2:**
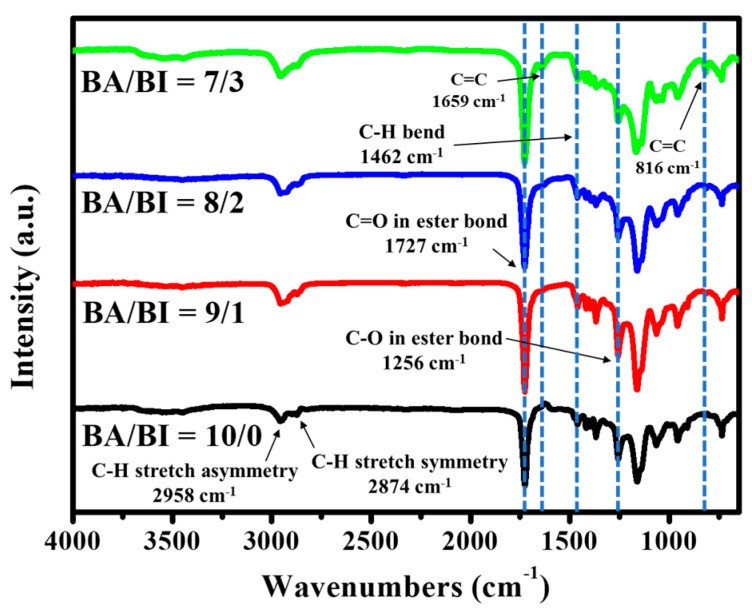
FT-IR spectra of the PBABI copolyesters.

Figure 2 shows the FT-IR spectra of PBABI copolyesters, for which the absorption peaks associated with the asymmetry and symmetry C–H stretch have been identified at 2958 and 2874 cm^−1^, respectively. The stretching vibration of the C=O of the ester bond was observed at 1727 cm^−1^, a C–H bend absorption peak was seen at a value of 1,462 cm^−1^, and a band in 1256 cm^−1^, related to the C–O of the ester bond. The most significant peak was identified and observed at 1659 and 816 cm^−1^, which was attributed to the stretching vibration of the C=C in the IA. The intensity of these two absorption peaks increased as the concentration of IA increased, demonstrating that the IA molecule was successfully copolymerized into the main-chain of the PBABI copolyesters. Moreover, the C=C bond of IA was protected by a radical inhibitor, 4-methoxyphenol, even through melt polymerization at a temperature of 230 °C, with further UV curing, to control the hardness of the PBABI copolyesters. This aligns with the results from Tang and his colleague [62], who found that IA-based polyester could form a cross-linked structure, through C=C of IA, using methyl methacrylate (MMA) as an initiator to improve the mechanical property, after irradiation under UV light; but, fumarate and maleate-based polyesters could not be induced by the UV curing process.

The results of gel permeation chromatography (GPC) analysis are shown in Table 2, and the PBABI copolyesters were dissolved in HFIP, for further measurement. The I.V. values of PBABI copolyesters in phenol and tetrachloroethane cosolvents (50/50, wt %) were estimated at 0.98, 1.02, 1.14, and 1.22 for BA/BI = 10/0, 9/1, 8/2, and 7/3, respectively [63]. Meanwhile, the *M*_n_ between 16,696 to 35,171 g·mole^−1^ indicated that the BA/BI = 9/1 had a better reactivity than other BA/BI ratios, under the same operating conditions. The *M*_w_ had more significant values from 29,280 to 178,838 g·mole^−1^, implying that the PBABI copolyesters had more massive globule structures because they could form partial cross-linking networks via the EDTA. The polydispersion index (PDI) ranged from 1.754 to 7.123, due to the more massive *M*_w_, resulting from a partially cross-linked structure forming the network architecture.

**Figure 3:**
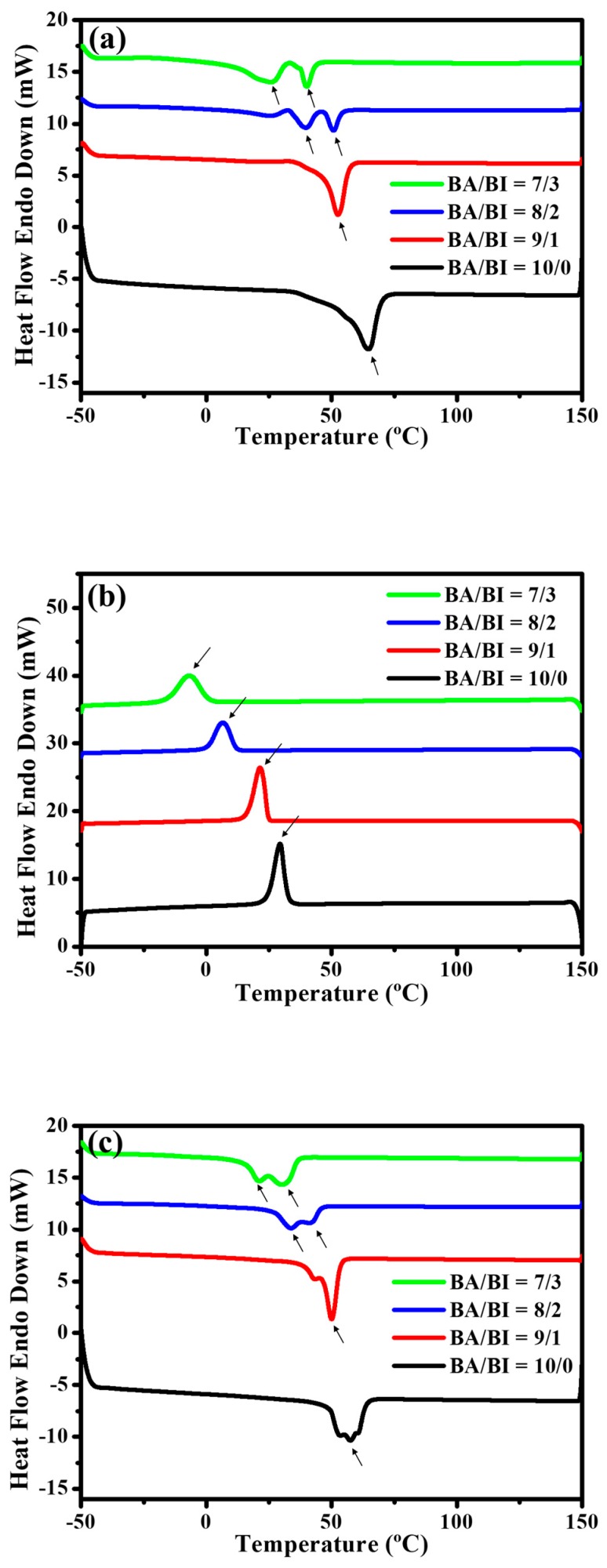
Differential Scanning Calorimetry (DSC) trace of the PBABI copolyesters. (**a**) Heating curves in the first cycle, (**b**) cooling curves in the first cycle, and (**c**) heating curves in the second cycle.

For the thermal behavior, the first cycle Figure 3a heating and Figure 3b cooling curve, and second cycle Figure 3c heating curve was run in DSC so that the crystallization in PBABI copolyesters could be observed during the cooling procedures, and the melting behavior could be seen in the subsequent reheating at 10 °C·min^−1^. The *T*_m_ decreased as IA concentration increased. Furthermore, the *T*_m_ peaks tended to separate into two peaks with IA concentrations above 20 mole%, in both the first and second cycle heating procedures, suggesting that AA and IA might form separate packing routines.

Competition for reactivity with 1,4-BDO was also observed in the copolymerization reaction. The ∆*H*_m_ decreased as IA increased, but the highest ∆*H*_m_ of the PBABI copolyesters was obtained at BA/BI = 9/1, at a value of 60.3 mJ·mg^−1^, caused by the more considerable molecular weight, to assist the molecular chain in packing well, into the crystal region. The thermal properties are listed in Table 3. In the first cycle cooling curve, the heating crystallization peak (∆*H*_hc_) was observed clearly at all BA/BI ratios, suggesting that PBABI copolyesters were easy to pack in the ordered phase [51]. The melting crystallization peak of the BA/BI was found in a single sharp peak around 20–30 °C, at ratios of 10/0 and 9/1. When the IA concentration was increased to 20 mole%, a broader crystallization peak was obtained, implying that greater IA concentration could hinder the molecular chain from packing into the ordered state, and disrupt the crystallization region of the AA-rich domain. In Figure 3c, the *T*_m_ was observed to be around 21.1–57.5 °C, and the melting peak (∆*H*_m_) appeared with a broader distribution at a ratio of BA/BI = 10/0 (neat PBA). After removing the thermal history, the melting peak of the PBABI copolyesters was shown to be in a continuous double peak, demonstrating that a competitive effect might exist in the crystallization zone of AA and IA, within the PBABI copolyesters, during melt polymerization.

**Figure 4:**
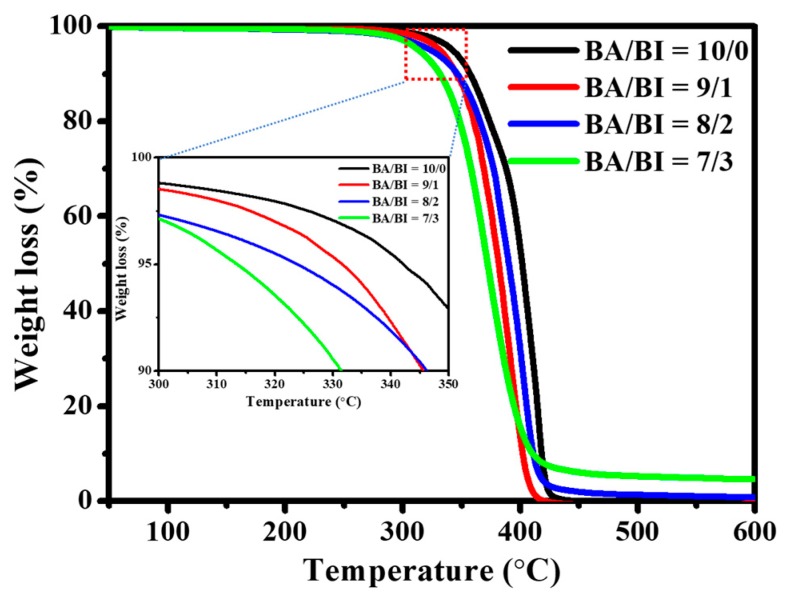
TGA curves of PBABI copolyesters.

The TGA curve is shown in Figure 4, and the 5 wt % weight loss temperature (*T*_d-5%_) of the PBABI copolyesters occurred around 313.6–342.1 °C, indicating that all synthesized PBABI copolyesters had excellent thermal stability since the *T*_d-5%_ of all PBABI copolyesters was over 300 °C. The thermal stability of the PBABI copolyesters decreased slightly, as the IA concentration increased, as can be seen in Table 3. Compared to the DSC results, all PBABI copolyesters displayed lower *T*_d-5%_ and *T*_m_ values, than a neat PBA with EDTA of 0.1 mole%. Hence, an increase in the IA concentration could not improve the thermal stability of the PBABI copolyesters, as a result of the disruption of the crystallization region.

**Figure 5:**
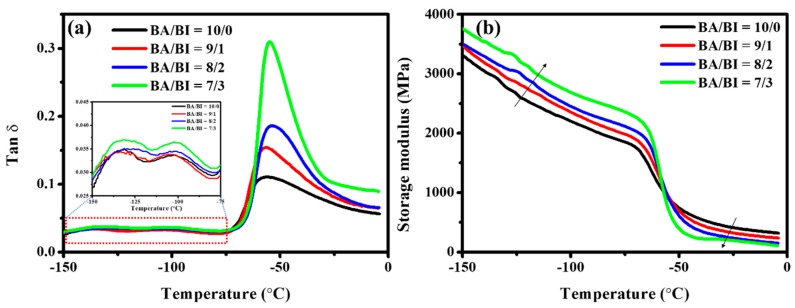
(**a**) Tan δ and (**b**) storage modulus of the PBABI copolyesters, via the dynamic mechanical analyzer.

Figure 5 shows the viscoelastic curves of the PBABI copolyesters. The second-order transition (*T*_g_) was not detected in the DSC measurement but was observed clearly via DMA, due to the temperature-range setting. The *T*_g_ of the PBABI copolyesters, obtained via DMA, maintained a stable value between −53.6 and −55.9 °C, as the IA concentration increased, as shown in Figure 5a. In contrast, the *T*_g_ was not affected by an increase in IA concentration with EDTA. The EDTA played an essential role in preserving the stability of the disordered region, even when the IA concentration was higher, due to the double-bond within IA, which maintained the amorphous zone. The β- and γ-relaxation were also measured around −100 and −130 °C, which was associated with the motion of the R-C=CH_2_ group, within IA, and the motion of the R-CH_2_ group within the aliphatic main-chain [64]. An increase in the storage modulus (*E*’) was observed below the *T*_g_ (glassy state) in Figure 5b, indicating that the molecular chain movement was fixed, and the physical property was dominated by raw chemicals in the glassy state, resulting from a more rigid double-bond within the IA. The transition behavior of E’ appeared near *T*_g_; *E*’ decreased when the temperature was raised above *T*_g_ (rubbery state), because the double-bond within IA could be induced to disturb and damage the main-chain molecules’ symmetry and regularity in the amorphous regime, to decrease the *E*’ in the rubbery state [51].

**Figure 6:**
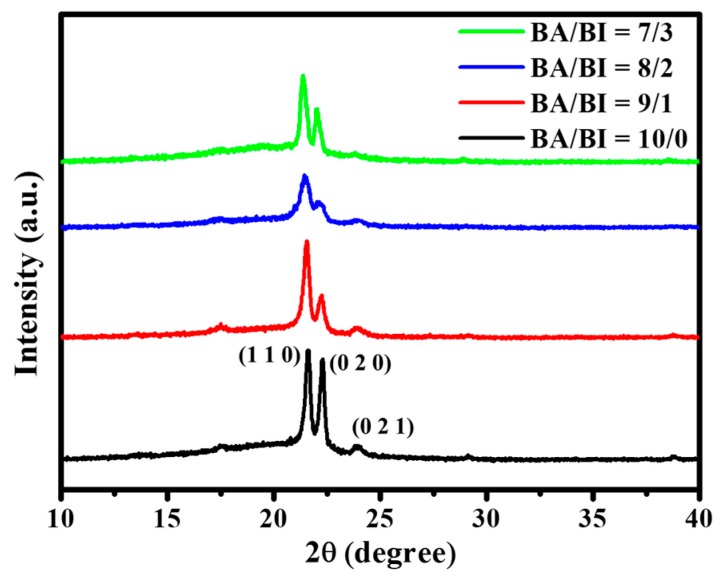
X-ray diffraction of the PBABI copolyesters.

The X-ray diffraction (XRD) of PBABI copolyesters in the 2θ of 10–40°, is shown in Figure 6. The XRD patterns of the PBABI had similar patterns to that of the PBA (BA/BI = 10/0), with a BI ratio of 30 mole%, demonstrating that the existence of the BI unit did not affect the crystal behavior of the BA unit. This was also observed in the poly(butylene succinate-*co*-butylene itaconate) system [65]. The feature peak of XRD was carried out at the 2θ values of 21.61°, 22.31°, and 23.96°, for the (110), (020), and (020) crystal lattices, respectively, which were associated with the α-phase of the neat PBA [38,66]. The (110) and (020) crystal lattices decreased as the IA concentration increased to BA/BI = 8/2; however, the intensity grew when the concentration increased to BA/BI = 7/3, implying that IA could participate in and enhance the chain packing into the ordered state, to increase the d-spacing. The percentage crystallinities of synthesized polyesters determined using XRD are listed in Table 3. PBABI copolyesters displayed crystallinity values within the range of 34.1% to 39.4%. Generally, the crystallinities of the PBABI copolyesters did not exceed 40%, which specified that the crystal formation was favored by the overall van der Waals interaction and by EDTA, to form cross-linked polyesters. PBABI copolyesters displayed an increase of chain flexibility with increasing BI content. This increased chain flexibility might have promoted a more-effective rearrangement of the polymer chains, permitting van der Waals interactions to contribute a more significant degree towards the crystalline domains formation, and might have increased the crystallinity. Generally speaking, the calculated crystallinities obtained via DSC were lower than those from XRD results; although, there were similar trends, suggesting a good correlation between the two measurements. In the NMR results in Figure 1, the concentration of the C=C bond of IA, within the PBABI copolyesters was maintained near 50%, in each ratio of the copolyesters, implying that 50% C=C of IA might be converted into saturated C–C, to support the cross-linking agent in forming a 3D network structure. Brännström et al. studied the poly(butylene itaconate-*co*-butylene succinate) (PBIBSu) system [50] via FT-IR analysis, which indicated the degree of curing of the PBIBSu copolyesters could be located around 50%–75%, with various ratios of IA and SuA. They hypothesized that the IA is not reactive enough under melt polymerization and the conversion of C=C in cross-linking operation could be achieved at 75%. At a 7/3 BA/BI ratio, the preserved C=C concentration was maintained at 15.01%, and the intensity of XRD increased in the (110) and (020) crystal lattices. Hence, staying near a 15% ratio for the C=C concentration of IA was critical for enhancing the unsaturated C=C to saturated C–C, to form a higher partial network architecture, which could decrease the main-chain motion to make the molecular chain easy to pack into the ordered state, thereby, increasing the crystal region. Furthermore, the (021) crystal lattice disappeared in the BI unit of 30 mole%, due to a higher degree of cross-linking.

**Figure 7:**
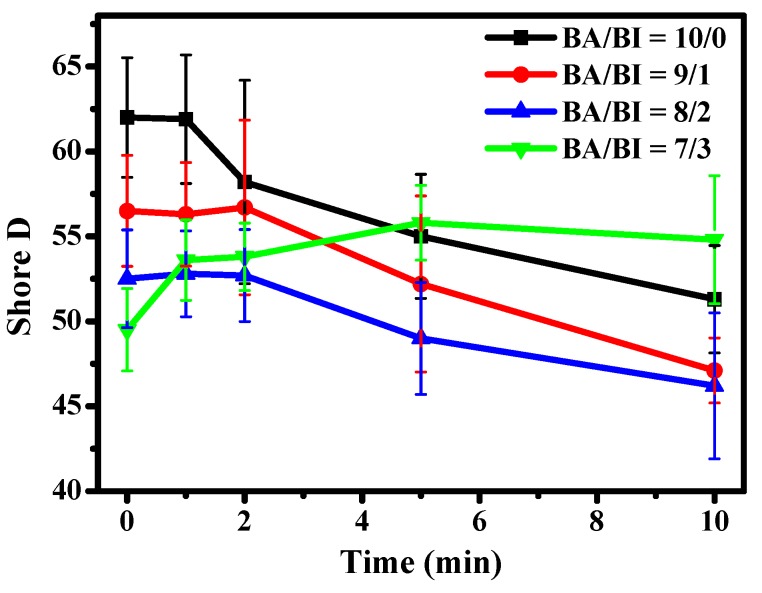
Shore D values at different UV curing times of the PBABI copolyesters.

Shore D as a function of UV curing time of the PBABI copolyesters was measured; shown in Figure 7. The highest hardness was observed at a ratio of BA/BI =10/0. The Shore D of synthesized PBABI copolyesters decreased as the IA concentration increased, due to the disruption of the crystal region. However, the Shore D values decreased gradually, when UV curing time through ultraviolet irradiation increased to more than 1 min, at a ratio of BA/BI = 10/0. At BA/BI = 9/1 and 8/2, the Shore D increased slightly, within 2 min, and then showed a smooth decline after 2 min of UV curing. It was notable that the Shore D of BA/BI = 7/3 increased within 5 min, during UV curing, which was attributed to the effectiveness of the C=C, in IA concentration, with regard to enhancing the degree of partial cross-linking, to improve the hardness of the PBABI copolyesters. The Shore D decreased slightly, after a UV curing time of 10 min, due to disruption of the structure from a higher thermal energy with the UV–Vis light. The results indicated that the cross-linking behavior of IA, occurring at BA/BI = 7/3, was triggered by the post-polymerization modification of the UV curing operation, resulting from sufficient C=C in IA concentration, at a value of 15 mole%, for cross-linking. The cured IA-based copolyesters were observed to enhance the *T*_g_ and storage modulus, suggesting that the C=C of IA could improve the thermal and mechanical characteristics, through UV curing procedures [50,67]. All specific Shore D values have been summarized in Table 4.

**Figure 8:**
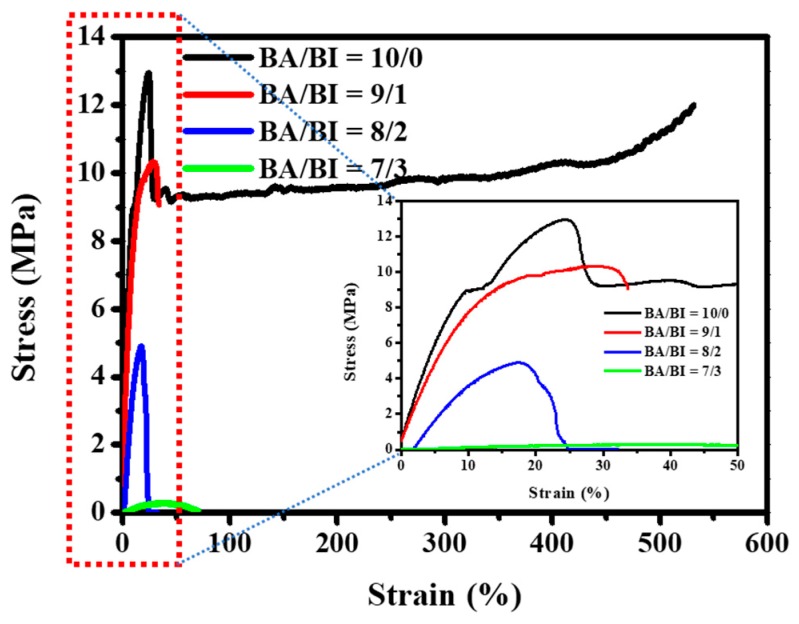
The stress–strain curves of the PBABI copolyesters.

Stress–strain measurements were performed to investigate the mechanical properties, including the yield strength, elongation at break, and Young’s modulus of the PBABI copolyesters. Those results were extracted from Figure 8 and are summarized in Table 5. In the tensile test, the macroscopic deformation of BA/BI = 10/0 (pure PBA) was examined at the highest strength (13.205 MPa) and elongation at break (~ 531.755%). All of the mechanical properties decreased and displayed a brittle characteristic when the IA ratio increased above 10 mole%. The yield strength, elongation, and Young’s modulus in BA/BI = 9/1 are shown for 9.328 MPa, 33.665%, and 94.093 MPa at room temperature measurement, respectively. Panic et al. [67] studied whether an increase in the length and concentration of the itaconate ester group could worsen mechanical properties and hinder the packing behavior of the molecular chain. The tensile test could not be measured accurately at BA/BI = 8/2 and 7/3, because their *T*_m_ values were observed at 33.8 and 21.1 °C, respectively, which was too close to or below RT (25 °C). Since BA/BI = 8/2 and 7/3 exhibited soft and viscose properties, respectively, the tensile test could not be performed at RT, through a universal tension machine. The real stress–strain curve could be tested at a lower temperature, to obtain the unique mechanical properties related to the storage modulus, implying that the PBABI copolyesters in low-temperature conditions might manifest an excellent mechanical behavior.

**Figure 9:**
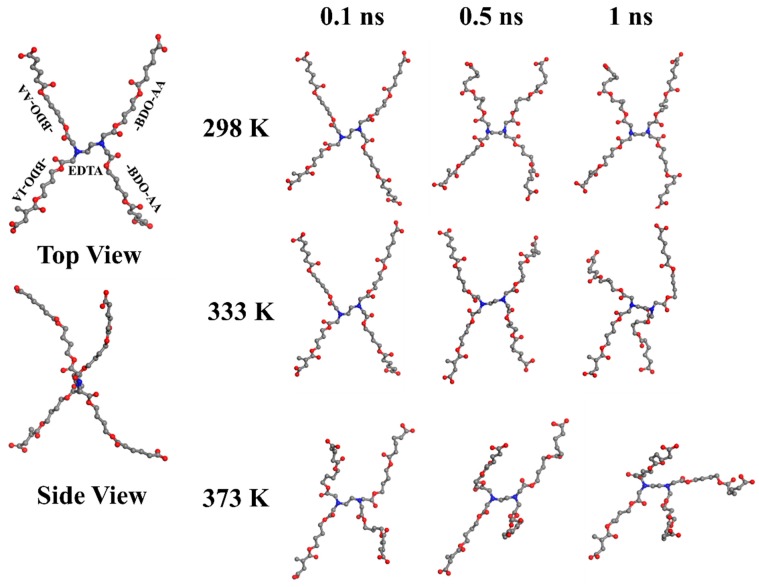
The structural deviation of the node point (EDTA-BDO-AA/IA) at various temperatures. (Color key—gray: carbon, red: oxygen, and blue: nitrogen, white: hydrogen (emitted)).

A simulation model has been designed and constructed with a center molecule of the EDTA, to investigate the rigidity of EDTA as a node within PBABI copolyesters. The EDTA has four COOH groups, which connect with four 1,4-BDOs with OH groups, to extend the length of the molecular chain, and then link to the three AA units and one IA unit, for our artificial initial geometrical conformation (as denoted by EDTA-BDO-BA/BI). This model for the BA/BI ratio was associated with a ratio of 75/25. The geometric conformation for the EDTA-BDO-BA/BI model was built in a tetrahedral shape. In Figure 9, the tetrahedral structure was maintained well at 298 K, after 1 ns molecular dynamics (MD) simulation, indicating that EDTA played a crucial role in supporting the stereo conformation. When the temperature increased, the stereo conformation became distorted and twisted, but the tetrahedral architecture was preserved by the central formation of the EDTA molecule, indicating that EDTA was not a sufficiently flexible or rigid molecule, and exhibited a semi-rigid property to play a node role within the cross-linking network.

**Figure 10:**
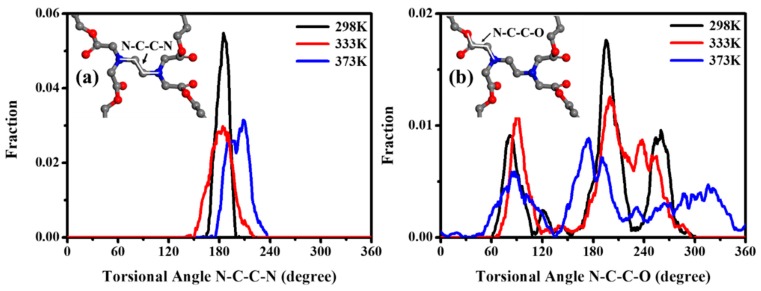
The torsional angle distribution of (**a**) N–C–C–N within the EDTA and (**b**) N–C–C–O between the EDTA and the 1,4-BDO.

The torsional angle distribution of N–C–C–N, within the EDTA and of N–C–C–O between the EDTA and the 1,4-BDO, as a function of time, were calculated at various temperatures, including 298 K, 333 K, and 373 K (as shown in Figure 10). The torsional angle of N–C–C–N was seen at 180° with a sharp peak, demonstrating that the center of EDTA between the two nitrogen atoms tended to form the coplanar and trans-conformation, even at higher temperatures of 333 K and 373 K [68]. Meanwhile, the torsional angle of N–C–C–O was maintained at 70°, 180°, and 250°, implying the geometric architecture exhibited a tetrahedral conformation. As the temperature increased, the peaks of N–C–C–O distribution stayed around 70°, 180°, and 250°, with a broader distribution, implying that the stereo conformation of tetrahedral shapes was distorted by thermal treatment, but was not disrupted into a disordered state [69]. In the process, EDTA as a cross-linking node tended to represent a semi-rigid molecule. When the EDTA’s semi-rigid characteristic was investigated, the chain motion near the EDTA could be restricted to form a nucleation site, which could make the main-chain near EDTA easier to pack into the ordered state to form crystal regions. In other words, the molecular chain linked with EDTA of PBABI copolyesters, could be rotated to reach a suitable steric conformation, which could be stacked into a requested state, as a result of the semi-rigid properties of the EDTA as a node.

### 3.2. The Effect of Varying EDTA Concentrations at BA/BI = 8/2 of PBABI Copolyesters

**Figure 11:**
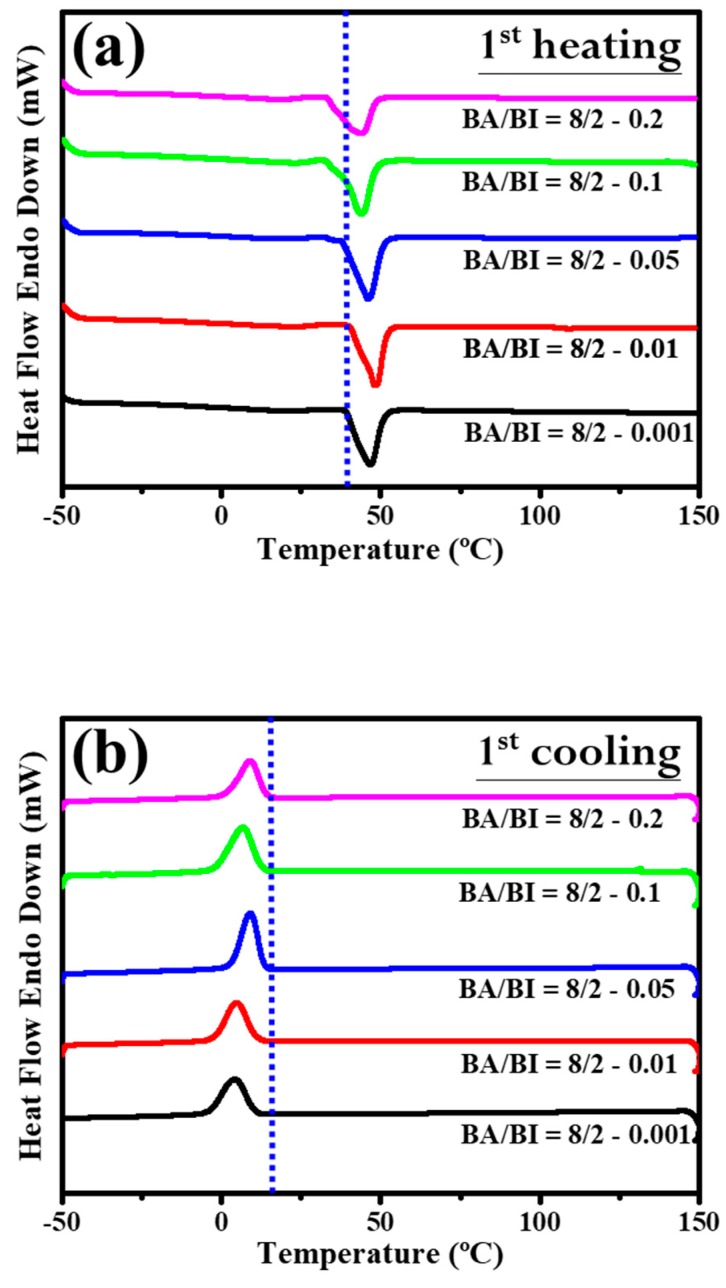

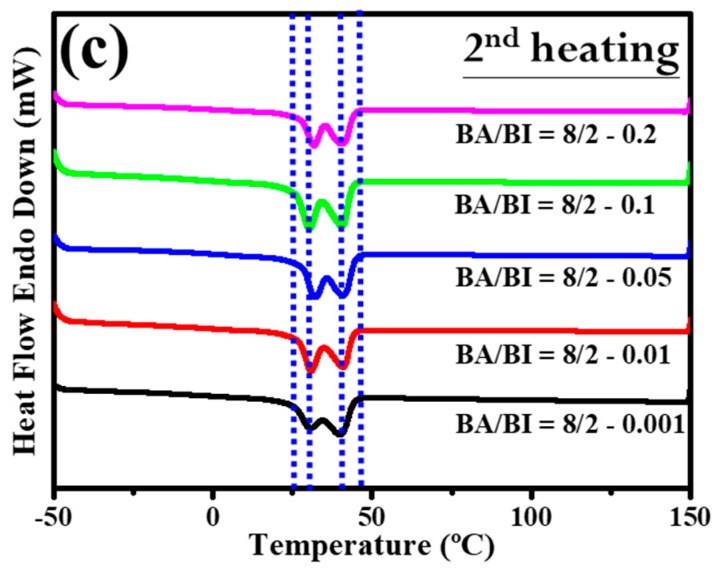
DSC trace of the PBABI copolyesters with different concentrations of EDTA. (**a**) Curves of heating in the first cycle, (**b**) curves of cooling in the first cycle, and (**c**) curves of heating in the second cycle.

Section 3.2 discussed the role of the EDTA concentration with the BA/BI of the PBABI copolyesters, supported by measurements of the thermal behavior and mechanical properties. Figure 11 shows the DSC trace for the PBABI copolyesters, to investigate the thermal response. In the first cycle of the heating procedure for DSC measurement in Figure 11a, the onset of *T*_m_ tended to lower the temperature as the EDTA concentration increased from 0.001 to 0.2 mole%, and all melting point peaks with different EDTA concentrations shifted to the left. The results confirmed that the thermal property was associated with the EDTA concentration, and the lowest onset temperature of *T*_m_ was located at an EDTA value of 0.2 mole%. The crystallization of each ratio of the PBABI copolyesters occurred from first cooling (as shown in Figure 11b). Crystallization was initiated at a higher temperature with an EDTA concentration of 0.2 mole%, and also had a larger enthalpy of crystallization (∆*H*_c_), implying the crystallization behavior was correlated with the amount of EDTA. The aliphatic molecular chain could be driven to pack into an ordered state, due to the limiting influence that a higher EDTA concentration had on the main-chain movement. The subsequent reheating procedure was displayed in Figure 11c, and the thermal history of the PBABI copolyesters was eliminated to precisely measure the *T*_m_ and ∆*H*_m_. The onset temperature and peak position of the melting point obtained similar curves, and the peak melting point was divided into two continuous peaks, representing the competitive effect between the AA and the IA, in the crystallization region. Additionally, the ∆*H*_m_ (as shown in Table 6) had a significant bell-shaped trend—∆*H*_m_ increased when EDTA concentration increased from 0.001 to 0.05 mole%, and then decreased at an EDTA ratio of 0.2 mole%. The results indicated that ∆*H*_m_ increased gradually as EDTA concentration increased, when there was a small amount of EDTA. However, more EDTA concentration as a node could decrease the freedom of the molecular chain, to hinder backbone packing into a crystal regime, thus, decreasing the enthalpy of crystallization.

**Figure 12:**
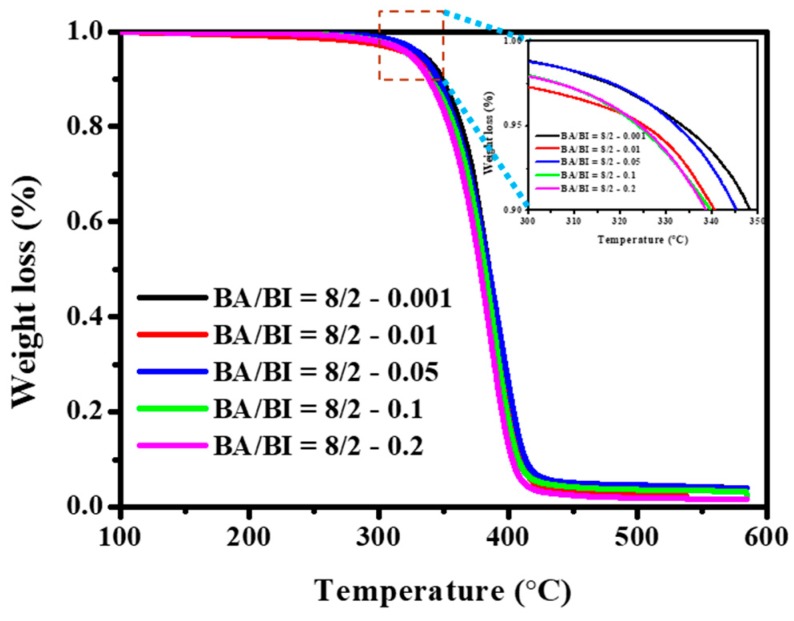
Thermogravimetric analysis (TGA) curve of the PBABI copolyesters with different concentrations of EDTA.

Figure 12 displays the TGA curves of the PBABI copolyesters, with varying concentrations of EDTA. Results suggest that the *T*_d-5%_ of the PBABI copolyesters decreased slightly between 333.4 to 323.7 °C, as the EDTA ratio increased, implying that using a higher EDTA concentration to enhance the degree of partial cross-linking was not suitable for improving the thermal degradation temperature of the PBABI copolyesters, since it disrupted crystallization.

**Figure 13:**
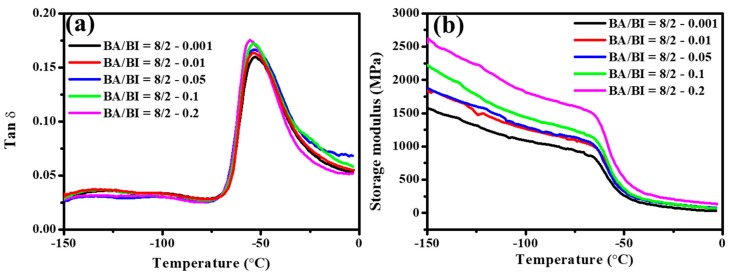
(**a**) Tan δ and (**b**) storage modulus of the PBABI copolyesters at different concentrations of EDTA.

Figure 13 illustrates the DMA analysis of Tan δ and storage modulus in a ratio of BA/BI = 8/2 of PBABI copolyesters, at different concentrations of EDTA. The Tan δ of PBABI copolyesters decreased slightly at higher concentrations of EDTA, and the *T*_g_ values were located around −54.5 ± 1.6 °C. The largest storage modulus was observed at an EDTA ratio of 0.2 mole%, suggesting that the PBABI copolyesters in both glassy and rubbery states tended to be harder, due to the relatively higher proportion of EDTA. A greater concentration of EDTA played a node role within copolyesters to form a tighter and well-dispersed 3D network architecture, which could improve the hardness of the PBABI copolyesters.

Thermal property measurements are outlined in Table 6. *T*_g_ was observed between −52.8 to −56.1 °C, with an increase in the EDTA ratio. At an EDTA ratio of 0.05 mole%, *T*_c_ was reached at a temperature of 9.3 °C, indicating it could crystallize at a higher temperature than other concentrations of EDTA, which was evident in the higher ∆*H*_c_ value of −43.8 mJ·mg^−1^. The DSC curve of T_m_ in the reheating procedure had a similar trajectory in the two separate peaks around 30 and 40 °C, but the ∆*H*m was slightly different. It is interesting that the most significant ∆*H*_m_ value occurred at an EDTA ratio of 0.05 mole% and then decreased at 0.1 mole%, implying that the optimal concentration of EDTA might be around 0.05–0.1 mole%.

**Figure 14:**
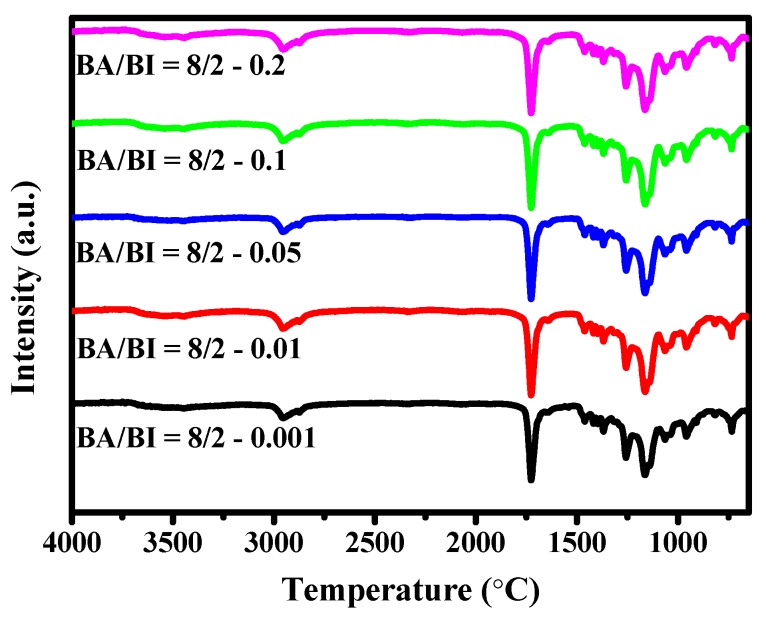
FT-IR spectra of the PBABI copolyesters with different concentrations of EDTA.

The absorption peak of FT-IR spectra in the PBABI copolyesters at various ratios of EDTA are displayed in Figure 14. It is not surprising that the FT-IR curve of each ratio of EDTA in BA/BI = 8/2 exhibited similar trends and absorption intensities, because the EDTA concentration was too low to examine the feature peak in FT-IR spectra. All absorption peak positions could be referred to Figure 2.

**Figure 15:**
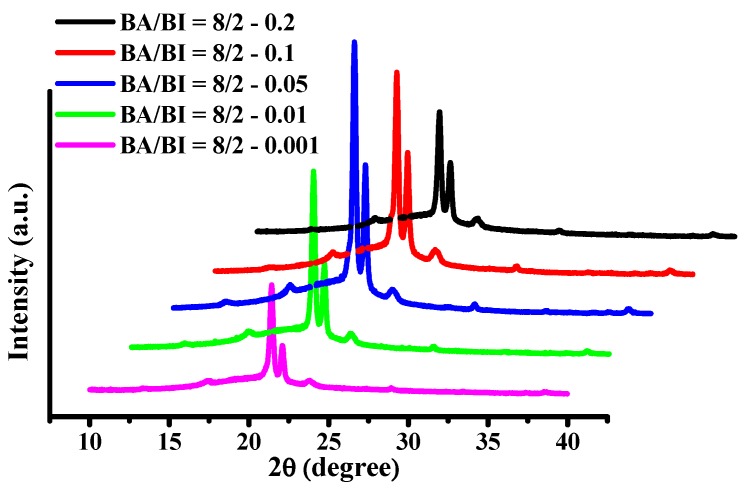
X-ray diffraction (XRD) of BA/BI = 8/2 of the PBABI copolyesters with different concentration of EDTA.

The XRD patterns for BA/BI = 8/2 of PBABI copolyesters at EDTA concentrations from 0.001 to 0.2 mole% are shown in Figure 15. The values of 2θ of BA/BI = 8/2 with EDTA of 0.001 mole% of PBABI copolyesters were identified at 21.46°, 22.13°, and 23.97°, which relates to the crystal lattices of (110), (020), and (020), respectively. The intensity of the crystal lattices at (110) and (020) increased with a ratio of EDTA between 0.001 to 0.05 mole%, which indicated that the degree of crystallization was increased by increasing the concentration of EDTA. The most considerable ∆*H*_m_ value was also observed at a ratio of 0.05 mole%. However, when the EDTA was increased to 0.1 and 0.2 mole%, the intensity of feature peaks of (110) and (020) decreased gradually, implying the crystal region was reduced as the EDTA increased. The results demonstrated that EDTA played a crucial role in controlling the enthalpy of crystallization. The lower concentration of EDTA might have decreased the chain rotation to enhance the crystal zone, and the crystal region became smaller with an increase in EDTA ratio to lower the ∆*H*_m_. Hence, our suggested optimal rate of EDTA as a cross-linking agent of PBABI copolyesters is between 0.05–0.1 mole%.

**Figure 16:**
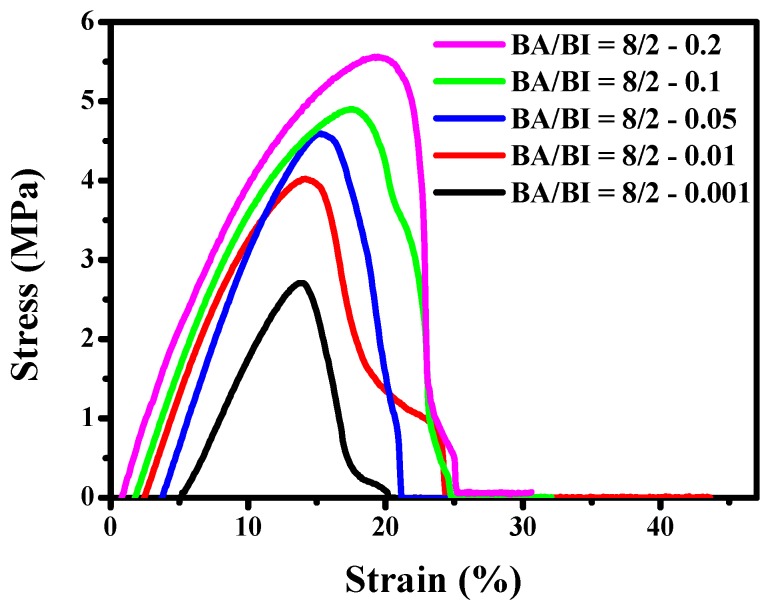
The stress–strain curves of the PBABI copolyesters at different concentrations of EDTA.

The stress–strain curves of the PBABI copolyesters at varying ratios of EDTA were measured as illustrated in Figure 16. The *T*_m_ of BA/BI = 8/2 of the PBABI copolyesters was carried out at 33.8 °C and tended to exhibit strong viscosity and soft properties. Hence, the dumb-bell testing sample of the PBABI copolyesters in the tensile test, could not accurately reflect the real stress value at room temperature. However, the trend in stress deviation increased with an increase in the EDTA concentration, which could be attributed to the higher partial degree of cross-linking. Fisher et al. [70] studied poly(propylene fumarate) with two initiators, bis(2,4,6-trimethyl benzoyl) phenyl phosphine oxide and monoacylphosphine oxide, after UV–Vis irradiation, and found that the 3D network structure might efficiently enhance the tensile modulus with different concentrations and types of cross-linking agents.

## 4. Conclusions

A series of PBABI copolyesters with varying ratios of EDTA were successfully synthesized through melt polymerization and identified using ^1^H NMR. In DSC measurements, the *T*_m_ and *T*_hc_ were observed around 21.1 to 57.5 °C and −6.7 to 29.5 °C. The *T*_g_ obtained through DMA measurement was observed around −53.6 to −55.8 °C, and was not affected by the concentration of IA. IA concentration increased alongside a decrease in thermal properties, which could be attributed to the disruption of the crystal region. In the XRD pattern, IA might have participated in enhancing chain packing into an ordered state, to increase the d-spacing. The tensile test could not offer accurate measurements at BA/BI = 8/2 and 7/3, because the *T*_m_ was either too close to (8/2) or below RT (7/3). The Shore D of BA/BI = 7/3 increased after 5 min of UV curing, due to the effect of a sufficient concentration of IA. In BA/BI = 8/2 at various concentrations of EDTA, there was a significant bell-shaped trend. The ∆*H*_m_ increased as the EDTA concentration increased from 0.001 to 0.1 mole%, and then decreased at an EDTA ratio of 0.2 mole%. The higher concentration of EDTA played a node role within copolyesters to form tighter and well-dispersed 3D network architectures, which could enhance the hardness of the PBABI copolyesters. The recommended optimal ratio of EDTA as a cross-linking agent of BA/BI = 8/2 of PBABI copolyesters might fall within a range of 0.05–0.1 mole%. Stress increased with an increase in the EDTA concentration, which was attributed to the higher partial degree of cross-linking. Finally, the unique *T*_m_ of the PBABI copolyesters could be adapted for applications to reinforce 3D air mesh fabric. Moreover, the advantages of the PBABI copolyesters could offer lightweight, breathable properties in 3D smart textiles and customizable and controllable hardness, through UV curing procedures.

## Figures and Tables

**Table 1:** The composition of the C=C bond of itaconic acid within PBABI copolyesters, chemical shifts (ppm), and the integral ratio (the value in brackets) of ^1^H NMR spectra for PBABI copolyesters.

Item	Feed Ratio	Calculated Ratio	H1	H2	H3	H4	H5	H6	H7	H8
BA/BI = 10/0	0	0	1.838 (1.004)	1.929 (0.992)	2.015 (0.028)	2.605 (1.000)		4.361 (1.001)		
BA/BI = 9/1	10	4.51	2.169 (0.997)	2.231 (0.992)	2.545 (0.015)	2.922 (1.000)	3.948 (0.031)	4.675 (1.024)	6.343 (0.02)	6.942 (0.02)
BA/BI = 8/2	20	11.41	2.015 (0.983)	2.101 (0.828)	2.408 (0.019)	2.793 (1.000)	3.794 (0.091)	4.547 (1.039)	6.224 (0.039)	6.814 (0.052)
BA/BI = 7/3	30	15.01	1.841 (1.000)	1.923 (0.944)	2.237 (0.02)	2.579 (1.000)	3.632 (0.125)	4.376 (1.101)	6.044 (0.075)	6.626 (0.047)

**Table 2:** Intrinsic viscosity (I.V.), *M*_n_, *M*_w_, and *M*_w_/*M*_n_ of gel permeation chromatography with hexafluoroisopropanol (HFIP) as the solvent for the PBABI copolyesters.

Item	^#^ I.V. (dl·g^−1^)	*M*_n_ (g·mole^−1^)	*M*_w_ (g·mole^−1^)	*M*_w_/*M*_n_ (PDI)
BA/BI = 10/0	0.98	16,696	29,280	1.754
BA/BI = 9/1	1.02	35,171	89,571	2.547
BA/BI = 8/2	1.14	28,323	113,520	4.008
BA/BI = 7/3	1.22	25,108	178,838	7.123

# is measured by Ubbelodhe viscometer.

**Table 3:** Thermal property of the PBABI copolyesters.

Item	* *T*_g_ (°C)	*T*_c_ (°C)	Δ*H*_c_ (mJ·mg^−1^)	*T*_m_ (°C)	Δ*H*_m_ (mJ·mg^−1^)	*T*_d-5%_ (°C)	^#^ X_c_ (%)
BA/BI = 10/0	−55.8	29.5	−46.1	57.5	40.4	342.1	39.4
BA/BI = 9/1	−55.9	22.7	−56.3	52.2	60.3	331.8	36.7
BA/BI = 8/2	−53.6	6.5	−32.5	33.8	32.0	323.9	34.1
BA/BI = 7/3	−54.8	−6.7	−33.2	21.1	32.2	313.6	37.9

* The *T*_g_ is measured by DMA. # the X_c_ is determined via XRD.

**Table 4:** The values of Shore D with standard deviation as a function of curing time for the PBABI copolyesters.

Cure Time (min)	BA/BI = 10/0	BA/BI = 9/1	BA/BI = 8/2	BA/BI = 7/3
0	62.0 ± 3.52	56.5 ± 3.27	52.5 ± 2.88	49.5 ± 2.43
1	61.9 ± 3.78	56.3 ± 3.06	52.8 ± 2.53	53.6 ± 2.37
2	58.2 ± 5.99	56.7 ± 5.14	52.7 ± 2.71	53.8 ± 1.99
5	55.0 ± 3.65	52.2 ± 5.28	49.0 ± 3.30	55.8 ± 2.20
10	51.3 ± 3.16	47.1 ± 1.91	46.2 ± 4.29	54.8 ± 3.77

**Table 5:** Mechanical properties of the PBABI copolyesters.

Item	Yield Strength (MPa)	Elongation (%)	Young’s Modulus (MPa)
BA/BI = 10/0	13.21 ± 1.24	531.76 ± 55.43	128.65 ± 12.23
BA/BI = 9/1	9.33 ± 1.03	33.67 ± 4.23	94.09 ± 8.37
BA/BI = 8/2	4.73 ± 0.45	26.24 ± 2.46	49.83 ± 2.44
BA/BI = 7/3 *	0.27 ± 0.07	71.04 ± 10.45	0.77 ± 0.12

* The *T*_m_ of BA/BI = 7/3 is below room temperature.

**Table 6:** Thermal properties of the PBABI copolyesters at different concentrations of EDTA.

Item	* *T*_g_ (°C)	*T*_c_ (°C)	Δ*H*_c_ (mJ·mg^−1^)	*T*_m_ (°C)	Δ*H*_m_ (mJ·mg^−1^)	*T*_d-5%_ (°C)
BA/BI = 8/2—0.001	−52.8	4	−37.6	30.6, 39.7	35.6	333.4
BA/BI = 8/2—0.01	−52.9	4.8	−39.4	30.9, 40.8	38.2	325.5
BA/BI = 8/2—0.05	−53.4	9.3	−43.8	32.0, 40.7	42.6	331.9
BA/BI = 8/2—0.1	−55.5	6.6	−40.8	30.5, 40.6	39.7	323.7
BA/BI = 8/2—0.2	−56.1	8.9	−39.3	31.9, 40.9	38.4	324.9

* The *T*_g_ is measured via DMA.

## References

[1] Rieckmann T., Völker S., Scheirs J., Long T.E. (2004). Poly(ethylene terephthalate) polymerization—Mechanism, catalysis, kinetics, mass transfer and reactor design. Wiley Series in Polymer Science.

[2] Chuah H.H., Scheirs J., Long T.E. (2004). Synthesis, properties and applications of poly(trimethylene terephthalate). Wiley Series in Polymer Science.

[3] Gallucci R.R., Patel B.R., Scheirs J., Long T.E. (2004). Poly(butylene terephthalate). Wiley Series in Polymer Science.

[4] Hall I., Ibrahim B. (1982). The structure and properties of poly(hexamethylene terephthalate): 1. The preparation, morphology and unit cells of three allomorphs. Polymer.

[5] Callander D.D., Scheirs J., Long T.E. (2004). Properties and applications of poly(ethylene 2,6-naphthalene), its copolyesters and blends. Wiley Series in Polymer Science.

[6] Bunn C.W. (1955). The melting points of chain polymers. J. Polym. Sci..

[7] Jovanovic D., Nikolic M., Djonlagic J. (2004). Synthesis and characterization of biodegradable aliphatic copolyesters with hydrophilic soft segments. J. Serbian Chem. Soc..

[8] Ozturk G.I., Pasquale A.J., Long T.E. (2010). Melt synthesis and characterization of aliphatic low-T_g_ polyesters as pressure sensitive adhesives. J. Adhes..

[9] Celli A., Marchese P., Sullalti S., Cai J., Gross R.A. (2013). Aliphatic/aromatic copolyesters containing biobased ω-hydroxyfatty acids: Synthesis and structure–property relationships. Polymer.

[10] Vert M. (2005). Aliphatic polyesters: Great degradable polymers that cannot do everything. Biomacromolecules.

[11] Velmathi S., Nagahata R., Takeuchi K. (2007). Extremely rapid synthesis of aliphatic polyesters by direct polycondensation of 1:1 mixtures of dicarboxylic acids and diols using microwaves. Polym. J..

[12] Sokolsky-Papkov M., Langer R., Domb A.J. (2011). Synthesis of aliphatic polyesters by polycondensation using inorganic acid as catalyst. Polym. Adv. Technol..

[13] Díaz A., Katsarava R., Puiggalí J. (2014). Synthesis, properties and applications of biodegradable polymers derived from diols and dicarboxylic acids: From polyesters to poly(ester amide)s. Int. J. Mol. Sci..

[14] Douka A., Vouyiouka S., Papaspyridi L.-M., Papaspyrides C.D. (2018). A review on enzymatic polymerization to produce polycondensation polymers: The case of aliphatic polyesters, polyamides and polyesteramides. Prog. Polym. Sci..

[15] Zia K.M., Noreen A., Zuber M., Tabasum S., Mujahid M. (2016). Recent developments and future prospects on bio-based polyesters derived from renewable resources: A review. Int. J. Biol. Macromol..

[16] Costa C.S.M.F., Fonseca A.C., Moniz J., Godinho M., Coelho J.F.J., Serra A.C. (2017). Going greener: Synthesis of fully biobased unsaturated polyesters for styrene crosslinked resins with enhanced thermomechanical properties. Express Polym. Lett..

[17] Robert T., Friebel S. (2016). Itaconic acid—A versatile building block for renewable polyesters with enhanced functionality. Green Chem..

[18] Mangeon C., Renard E., Thevenieau F., Langlois V. (2017). Networks based on biodegradable polyesters: An overview of the chemical ways of crosslinking. Mater. Sci. Eng. C.

[19] Farmer T.J., Comerford J.W., Pellis A., Robert T. (2018). Post-polymerization modification of bio-based polymers: Maximizing the high functionality of polymers derived from biomass: Post-polymerization modification of bio-based polymers. Polym. Int..

[20] Kumar S., Samal S.K., Mohanty S., Nayak S.K. (2018). Synthesis and characterization of itaconic-based epoxy resins. Polym. Adv. Technol..

[21] Toorkey R.F., Rajanna K.C., Prakash P.K.S. (1996). Curing of unsaturated polyester: Network formation. J. Chem. Educ..

[22] Sperling L.H., Klempner D., Sperling L.H., Utracki L.A. (1994). Interpenetrating polymer networks: An overview. Interpenetrating Polymer Networks.

[23] Roland C.M., Kobayashi S., Müllen K. (2013). Interpenetrating polymer networks (IPN): Structure and mechanical behavior. Encyclopedia of Polymeric Nanomaterials.

[24] Auras R., Lim L.-T., Selke S.E.M., Tsuji H. (2010). Poly(Lactic Acid): Synthesis, Structures, Properties, Processing, and Applications.

[25] Dorgan J.R., Braun B., Wegner J.R., Knauss D.M., Khemani K.C., Scholz C. (2006). Poly(lactic acids): A Brief review. Degradable Polymers and Materials.

[26] Kweon H. (2003). A novel degradable polycaprolactone networks for tissue engineering. Biomaterials.

[27] Izquierdo R., Garcia-Giralt N., Rodriguez M.T., Cáceres E., García S.J., Gómez Ribelles J.L., Monleón M., Monllau J.C., Suay J. (2008). Biodegradable PCL scaffolds with an interconnected spherical pore network for tissue engineering. J. Biomed. Mater. Res. A.

[28] Rodriguez E.D., Luo X., Mather P.T. (2011). Linear/network poly(ε-caprolactone) blends exhibiting shape memory assisted self-healing (SMASH). ACS Appl. Mater. Interfaces.

[29] Pal J., Sanwaria S., Choudhary A., Thakur K., Nandan B., Srivastava R.K. (2014). Thermally initiated trans-esterification in poly(ε-caprolactone) and its dependence on molecular weight. J. Polym. Environ..

[30] Hankermeyer C.R., Tjeerdema R.S., Ware G.W. (1999). Polyhydroxybutyrate: Plastic made and degraded by microorganisms. Reviews of Environmental Contamination and Toxicology.

[31] Zaheer M.R., Kuddus M. (2018). PHB (poly-β-hydroxybutyrate) and its enzymatic degradation. Polym. Adv. Technol..

[32] Xu J., Guo B.-H. (2010). Poly(butylene succinate) and its copolymers: Research, development and industrialization. Biotechnol. J..

[33] Gigli M., Fabbri M., Lotti N., Gamberini R., Rimini B., Munari A. (2016). Poly(butylene succinate)-based polyesters for biomedical applications: A review. Eur. Polym. J..

[34] Vandesteene M., Jacquel N., Saint-Loup R., Boucard N., Carrot C., Rousseau A., Fenouillot F. (2016). Synthesis of branched poly(butylene succinate): Structure properties relationship. Chin. J. Polym. Sci..

[35] Guo B., Chen Y., Lei Y., Zhang L., Zhou W.Y., Rabie A.B.M., Zhao J. (2011). Biobased poly(propylene sebacate) as shape memory polymer with tunable switching temperature for potential biomedical applications. Biomacromolecules.

[36] Minke R., Blackwell J. (1979). Polymorphic structures of poly(tetramethylene adipate). J. Macromol. Sci. Part B.

[37] Righetti M.C., Pizzoli M., Lotti N., Munari A. (1998). Crystallization kinetics and melting behavior of poly(butylene adipate), poly(butylene isophthalate) and their copolymers. Macromol. Chem. Phys..

[38] Woo E.M., Wu M.C. (2005). Thermal and X-ray analysis of polymorphic crystals, melting, and crystalline transformation in poly(butylene adipate). J. Polym. Sci. Part B.

[39] Liu F., Chi D.-Q., Na H.-N., Zhu J. (2018). Isothermal crystallization kinetics and crystalline morphologies of poly(butylene adipate-co-butylene 1,4-cyclohexanedicarboxylate) copolymers. Chin. J. Polym. Sci..

[40] Brännström S., Finnveden M., Johansson M., Martinelle M., Malmström E. (2018). Itaconate based polyesters: Selectivity and performance of esterification catalysts. Eur. Polym. J..

[41] Kumar S., Krishnan S., Samal S.K., Mohanty S., Nayak S.K. (2017). Itaconic acid used as a versatile building block for the synthesis of renewable resource-based resins and polyesters for future prospective: A review: Itaconic acid as building block for synthesis: A review. Polym. Int..

[42] Herrera R., Franco L., Rodríguez-Galán A., Puiggalí J. (2002). Characterization and degradation behavior of poly(butylene adipate-co-terephthalate)s: Poly(butylene adipate-co-terephthalate)s. J. Polym. Sci. Part Polym. Chem..

[43] Tomić S.L., Filipović J.M. (2004). Synthesis and characterization of complexes between poly(itaconic acid) and poly(ethylene glycol). Polym. Bull..

[44] Wibowo A.H., Makhnunah N., Irawati D., Purnawan C., Nurhayati N.D., Storz H. (2014). Synthesis and thermal-stability study of polybutylene itaconate modified with divinyl benzene and glycerol. Indones. J. Chem..

[45] Sousa A.F., Fonseca A.C., Serra A.C., Freire C.S.R., Silvestre A.J.D., Coelho J.F.J. (2016). New unsaturated copolyesters based on 2,5-furandicarboxylic acid and their crosslinked derivatives. Polym. Chem..

[46] Dai J., Ma S., Teng N., Dai X., Shen X., Wang S., Liu X., Zhu J. (2017). 2,5-furandicarboxylic acid- and itaconic acid-derived fully biobased unsaturated polyesters and their cross-linked networks. Ind. Eng. Chem. Res..

[47] Wu Y., Xie Q., Gao C., Wang T., Wang C. (2014). Synthesis and characterization of a novel aliphatic polyester based on itaconic acid. Polym. Eng. Sci..

[48] Pfeifer V.F., Vojnovich C., Heger E.N. (1952). Itaconic acid by fermentation with Aspergillus Terreus. Ind. Eng. Chem..

[49] Kuenz A., Krull S. (2018). Biotechnological production of itaconic acid—Things you have to know. Appl. Microbiol. Biotechnol..

[50] Brännström S., Malmström E., Johansson M. (2017). Biobased UV-curable coatings based on itaconic acid. J. Coat. Technol. Res..

[51] Gao C., Wang J., Han S., Hu Z., Liu Y. Copolymerization modification of poly(butylene itaconate). Proceedings of the AIP Conference.

[52] Blaurock Busch E.K. (2016). EDTA: Ethylene diamine tetra acetic acid—A review. Occup. Med. Health Aff..

[53] Rahman M.A., Won M.-S., Shim Y.-B. (2003). Characterization of an EDTA bonded conducting polymer modified electrode: Its application for the simultaneous determination of heavy metal ions. Anal. Chem..

[54] Tomaz V.A., Rubira A.F., Silva R. (2016). Solid-state polymerization of EDTA and ethylenediamine as one-step approach to monodisperse hyperbranched polyamides. RSC Adv..

[55] Nielsen L.E. (1969). Cross-linking–effect on physical properties of polymers. J. Macromol. Sci. Part C.

[56] Chen C.-W., Huang C.-I. (2015). Effects of intra/inter-molecular potential parameters, length and grafting density of side-chains on the self-assembling behavior of poly(3′-alkylthiophene)s in the ordered state. Polymer.

[57] Sun H., Ren P., Fried J.R. (1998). The COMPASS force field: Parameterization and validation for phosphazenes. Comput. Theor. Polym. Sci..

[58] Hoover W.G., Ladd A.J.C., Moran B. (1982). High-strain-rate plastic flow studied via nonequilibrium molecular dynamics. Phys. Rev. Lett..

[59] Nosé S. (1984). A unified formulation of the constant temperature molecular dynamics methods. J. Chem. Phys..

[60] Tang T., Moyori T., Takasu A. (2013). Isomerization-free polycondensations of cyclic anhydrides with diols and preparation of polyester gels containing *cis* or *trans* carbon double bonds via photo-cross-linking and isomerization in the gels. Macromolecules.

[61] Schoon I., Kluge M., Eschig S., Robert T. (2017). Catalyst influence on undesired side reactions in the polycondensation of fully bio-based polyester itaconates. Polymers.

[62] Tang T., Takasu A. (2015). Facile synthesis of unsaturated polyester-based double-network gels via chemoselective cross-linking using Michael addition and subsequent UV-initiated radical polymerization. RSC Adv..

[63] Moroni A., Havard T., Provder T. (1999). Characterization of polyesters and polyamides through sec and light scattering using 1,1,1,3,3,3-hexafluoro-2-propanol as eluent. Chromatography of Polymers.

[64] Arrighi V., McEwen I.J., Holmes P.F. (2004). Dielectric relaxations in poly(di-n-alkyl itaconate)s. Macromolecules.

[65] Liu Q., Zhou X.-M. (2015). Syntheses and physical characterization of biodegradable poly(butylene succinate-co-butylene itaconate) copolymers. J. Macromol. Sci. Part A.

[66] Wang H., Gao Z., Yang X., Liu K., Zhang M., Qiang X., Wang X. (2018). Epitaxial crystallization behavior of poly(butylene adipate) on orientated poly(butylene succinate) substrate. Polymers.

[67] Panic V.V., Seslija S.I., Popovic I.G., Spasojevic V.D., Popovic A.R., Nikolic V.B., Spasojevic P.M. (2017). Simple one-pot synthesis of fully biobased unsaturated polyester resins based on itaconic acid. Biomacromolecules.

[68] Jorgensen W.L. (1981). Pressure dependence of the structure and properties of liquid n-butane. J. Am. Chem. Soc..

[69] Chau P.-L., Hardwick A.J. (1998). A new order parameter for tetrahedral configurations. Mol. Phys..

[70] Fisher J.P., Timmer M.D., Holland T.A., Dean D., Engel P.S., Mikos A.G. (2003). Photoinitiated cross-linking of the biodegradable polyester poly(propylene fumarate). Part, I. Determination of network structure. Biomacromolecules.

